# Variations in the prevalence of caesarean section deliveries in India between 2016 and 2021 – an analysis of Tamil Nadu and Chhattisgarh

**DOI:** 10.1186/s12884-023-05928-4

**Published:** 2023-08-30

**Authors:** Varshini Neethi Mohan, P Shirisha, Girija Vaidyanathan, V R Muraleedharan

**Affiliations:** Department of Humanities and Social Sciences, Indian Institute of Technology Madras (IIT Madras), Chennai, 600 036 Tamil Nadu India

**Keywords:** Caesarean section, C-section delivery, India, NFHS-5, India, Private Sector, Inequality

## Abstract

**Background:**

The prevalence of C-sections in India increased from 17.2% in 2016 to 21.5% in 2021. This study examines the variations in C-section prevalence and the factors correlating to these variations in Tamil Nadu (TN) and Chhattisgarh (CG).

**Methods:**

Delivery by C-section as the outcome variable and several demographic, socio-economic, and clinical variables were considered as explanatory variables to draw inferences from unit-level data from the National Family Health Survey (NFHS-4; 2015-16 and NFHS-5; 2019-21). Descriptive statistics, bivariate percentage distribution, Pearson’s Chi-square test, and multivariate binary logistic regression models were employed. The Slope Index of Inequality (SII) and the Concentration Index (CIX) were used to analyse absolute and relative inequality in C-section rates across wealth quintiles in public- and private-sector institutions.

**Results:**

The prevalence of C-sections increased across India, TN and CG despite a decrease in pregnancy complications among the study participants. The odds of caesarean deliveries among overweight women were twice (OR = 2.11; 95% CI 1.95–2.29; NFHS-5) those for underweight women. Women aged 35–49 were also twice (OR = 2.10; 95% CI 1.92–2.29; NFHS-5) as likely as those aged 15–24 to have C-sections. In India, women delivering in private health facilities had nearly four times higher odds (OR = 3.90; 95% CI 3.74–4.06; NFHS-5) of having a C-section; in CG, the odds were nearly ten-fold (OR = 9.57; 95% CI:7.51,12.20; NFHS-5); and in TN, nearly three-fold (OR = 2.65; 95% CI-2.27-3.10; NFHS-5) compared to those delivering in public facilities. In public facilities, absolute inequality by wealth quintile in C-section prevalence across India and in CG increased in the five years until 2021, indicating that the rich increasingly delivered via C-sections. In private facilities, the gap in C-section prevalence between the poor (the bottom two quintiles) and the non-poor narrowed across India. In TN, the pattern was inverted in 2021, with an alarming 73% of the poor delivering via C-sections compared to 64% of those classified as non-poor.

**Conclusion:**

The type of health facility (public or private) had the most impact on whether delivery was by C-section. In India and CG, the rich are more likely to have C-sections, both in the private and in the public sector. In TN, a state with good health indicators overall, the poor are surprisingly more likely to have C-sections in the private sector. While the reasons for this inversion are not immediately evident, the implications are worrisome and pose public health policy challenges.

## Introduction

Caesarean section (C-section) deliveries, when medically justified, can be lifesaving for both mother and child [1]; when not strictly necessary, however, they can result in a number of short-term and long-term adverse health outcomes for both women as well as neonates such as maternal infection, uterine haemorrhage, infant respiratory distress, and hypoglycaemia [2]. Women who deliver via C-section after a prior caesarean delivery are more likely to discontinue breastfeeding as compared to those who deliver vaginally [3]. C-section deliveries are linked with longer hospital stays and higher out-of-pocket expenditures [4]. Additionally, they place an unnecessary strain on already scarce public health resources [5, 6].

The rise in the proportion of C-section deliveries across the world in general and in India in particular, has been the focus of several studies [7]. That C-section delivery rates in private facilities are, in general, far higher than those in public hospitals is of particular concern [8]. Evidence suggests that inequalities exist both between and within countries. A recent study analysed within-country wealth-related inequalities in 72 LMICs (Low- and Middle-Income Countries) and found that substantial subgroup inequalities exist [9]. There is a high geographical variability within India as well [10], with prevalence ranging from 5.2% in Nagaland to 60.7% in Telangana [11].

Several factors have been found to have an impact on C-section rates across the world and in India. These range from individual characteristics of the mother, such as age at delivery [12] and at marriage [10], obesity [13], education levels [14] and exposure to media [15]; and those of the child, such as birth order and the size of the child at birth [16]; to demographic and community-level factors such as caste [10, 15], place of residence [14, 16], wealth [17], the number of number of antenatal care centre visits [14] and whether the delivery happened in a private or a public hospital [10]. Some studies suggest that mothers’ preferences, either due to fear related to prolonged labour and vaginal delivery pain [18] or to beliefs in auspicious times [19] could also lead to delivery by C-section.

This paper analyses the new data from the latest round of the National Family Health Survey (NFHS-5–2019-21) [11] and compares it with data from NFHS-4–2015-16 [20]. It studies the trends in prevalence of C-sections, explores the factors that influence delivery by C-section, and lists those that significantly impact the odds of a woman delivering via a caesarean section. It brings out the contrast between two states in India with very different demographic and developmental features. In addition, it looks at wealth-related inequalities in delivery by C-section. Several interesting insights arise from these analyses.

In Section 1, we look at the trends in overall prevalence of C-sections in India between and NFHS-4 (2015-16) and NFHS-5 (2019-21). We choose Chhattisgarh and Tamil Nadu as states representing a significant contrast in terms of socio-economic and infrastructural characteristics and tabulate descriptive statistics of women who had live births in the five years preceding the last two rounds of NFHS. Section 2 lists the correlation of delivery by C-section with several explanatory variables, and Section 3 sets down the odds of a C-section delivery against a reference category for each significantly correlated explanatory variable. We find that whether delivery happened in a public-sector or a private-sector hospital has the most impact on C-section deliveries, and to analyse this further, Section 4 studies the prevalence of C-sections in public-sector and private-sector hospitals, and measures inequalities according to wealth.

## Methods

### Source of data

We used data from the fourth and fifth rounds of the National Family Health Survey (NFHS) conducted in 2015–2016 and 2019-21 respectively. NFHS is a nationally representative survey which generates data on population and health indicators, especially on maternal and child health indicators. This survey is conducted across all 29 states and union territories and is the Indian equivalent of a Demographic Health Survey (DHS). It is being conducted at regular intervals since 1992–93 under the stewardship of the Ministry of Health and Family Welfare and coordinated by the International Institute of Population Sciences (IIPS), Mumbai. The data provides state and national-level information on fertility, family planning, infant and child morbidity and mortality, maternal and reproductive health, nutritional status of women and children, and the quality of health services. The survey adopts a three-stage sample design for urban areas, and a two-stage sample design for rural areas. For urban areas, in the first stage, wards are selected. Census Enumeration Blocks (CEB) containing approximately 150/200 households are selected during the second stage, and the required number of households are then selected for the third stage using a systematic sampling technique. In most rural areas, the survey adopts a two-way sampling design where the villages are selected by probability proportional to size (PPS) sampling in the first stage while in the second stage, the required number of households are selected using systematic sampling. This ensures that the sample size is sufficient to carry out both state and national-level analysis.

A detailed description of the sampling design and survey procedure has been provided in the national report of the NFHS [11]. In NFHS-4 (2014-16), In NFHS-4 (2014-16), interviews were completed with 699,686 women with response rate of 92% (International Institute for Population Sciences (IIPS) & ICF, 2017). In NFHS-5 (2019-21), interviews were completed with 724,115 women, with a response rate of 97% [11] .

### Outcome variable

The outcome variable in our study is whether the woman had self-reportedly undergone a caesarean section, which was measured in a binary response: yes or no. The question that women were asked in the 4th and 5th round of NFHS was if “the baby was delivered by caesarean section, that is, did they cut your belly open to take the baby out?” Women who responded in the affirmative were classified as having “delivered by caesarean section”.

### Explanatory variables

Covariates were selected a priori based on a literature review [10, 12–17, 21–23].

1. Respondent’s caste (Scheduled Caste, Scheduled Tribe, Other Backward castes, Others),
2. Respondent’s religion (Hindu, Muslim, others),
3. Respondent’s place of residence (urban, rural),
4. Respondent’s educational level (no education, primary, secondary, and higher),
5. Respondent’s age at marriage (less than 18, 18 or above),
6. Respondent’s exposure to mass media (none, partial or full exposure), measured from the frequency of listening to the radio, reading newspapers/magazines, and watching television,
7. Number of Antenatal Care (ANC) visits (none, 1–3 visits, and 4 or more),
8. Respondent’s age (15–24, 25–34, and 35–49 years),
9. Size of the child at birth as perceived by the mother (average, small or large),
10. Respondent’s Body Mass Index (BMI) (underweight if < 18.5 kg/m^2^, normal if 18.5–24.9 kg/m^2^, and overweight if > 25 kg/m^2^),
11. Pregnancy complications were grouped as a binary response: “yes” (women who suffered from convulsions, swelling of body or legs, or had difficulty with daylight vision) and “no” (women who did not suffer from any complications during pregnancy),
12. Ever had a terminated pregnancy – this was operationalized as a binary variable into “yes” (women who had ever had an abortion, a miscarriage, or a stillbirth) and “no” (women who had a terminated pregnancy),
13. Birth order of the child (first, second, third and fourth or greater),
14. Wealth quintile (poorest, poor, middle, rich and richest),
15. Place of delivery (public - including government/municipality hospital, government dispensary, urban health centre or post, an urban family welfare centre, community health centre, block primary health centre, sub-centre or private - including private hospital/maternity home/clinic, non-government organization, and trust hospital/ clinic),

### Measures of socioeconomic position

Measuring socioeconomic status in low- and middle-income countries poses several challenges [24]. To overcome these, Filmer and Pritchett devised an Asset Index in 1998 [25]. The asset index is the most common and preferred index used to assess low- and middle-income countries and includes a list of household items such as TV, radio, refrigerator, building, toilet, etc. [26]. NFHS estimates the wealth quintile by assigning scores based on assets and housing characteristics. The score of each household asset is derived using Principal Component Analysis (PCA). The resulting asset scores are standardized against a mean of 0 and a standard deviation of 1. The scores of the household are then assigned to the residents of that household, after which the individuals are ranked according to their score in the household population. They are divided into five equal categories, each with 20% of the specimen population [27]. These quintiles are poorest (Q1), poor (Q2), middle (Q3), richer (Q4), and richest (Q5). To assess the magnitude of inequality in the coverage of health services across households from different economic strata, we have used the nationwide data on the wealth quintile provided by NFHS [11].

### Statistical analysis

Our study sample was defined as the most recent live birth by C-section in the five years preceding NFHS-4 (2015-16) and − 5 (2019-21). We used descriptive statistics to present the socio-demographic characteristics of women who gave birth to a live child in the preceding 5 years. To estimate percentages, the sample weight was used. Bivariate analyses were performed with various explanatory variables against the outcome variable of whether delivery was via C-section. This was done to identify covariates with which there is an association with the outcome. The level of significance in the correlation between the result and explanatory variables was tested using Pearson’s chi-square [28].

Using the factors identified as significant, multivariate logistic regression [29] was used to model the factors influencing the use of caesarean sections across India, Chhattisgarh, and Tamil Nadu. Statistical significance was declared at *p* < 0.05, and variables that were found to be statistically significant were included in the multivariate logistic regression model. The Variance Inflation Factor (VIF) demonstrated no multicollinearity among the chosen explanatory variables. The odds ratio (OR) was presented with a 95% confidence interval (CI). An odds ratio (OR) larger than 1 denoted that the probability of caesarean birth for that specific explanatory variable was higher than those of the reference category. Caesarean delivery was a binary variable coded as 0 for a vaginal delivery and 1 for a caesarean delivery.

We estimated the wealth-related inequality in the prevalence of C-sections among women in India, in Chhattisgarh (CG) and in Tamil Nadu (TN). The existing methods of absolute and relative inequality measures have certain limitations [30]. To overcome these limitations, Harper and Lynch came up with a set of more robust measures of inequality: the Slope Index of Inequality (SII), as an indicator of absolute inequality, and the Concentration Index (CIX) or the Relative Index of Inequality (RII), as an indicator of relative inequality [31]. SII is a weighted measure of inequality representing the absolute difference in estimated values between the most advantaged and most disadvantaged sections. While measuring the SII, linear regression of the dependent variable (intervention) over the wealth index (explanatory variable) gives a slope, which provides an absolute measure of the difference (in the intervention coverage) between the highest (score of 1) and the lowest (score of 0) values (which are, the richest and the poorest quintiles in our case) in the socioeconomic indicator rank. A positive value implies a pro-rich pattern, while a negative value shows pro-poor prevalence. CIX is related to the Gini coefficient, a well-known wealth/income concentration measure. Individuals are ranked according to their socioeconomic status on the x-axis, while cumulative intervention coverage is plotted on the y-axis. The distance between the curve and the diagonal denotes the concentration of wealth. It shows up to what extent an intervention is concentrated among the wealthiest or poorest [30]. A positive value implies that the coverage of interventions is pro-rich, i.e., the women from the higher wealth quintile have higher c-section rates. A negative value implies the prevalence of C-sections is higher among poorer women, children and households. Its value ranges from − 1 to 1 [32]. To bring out the differences between states, we grouped the lowest two quintiles as per national thresholds and classified them as “poor”, and grouped the upper three quintiles as “non-poor”, given that among the study participants in India (those who had given birth to a living child in the five years preceding the study), the distribution was then almost even (48% and 52% respectively) in NFHS-5. The contrast between Chhattisgarh and Tamil Nadu was stark, with 63% and 17% respectively classified as “poor” according to national standards (Table 1).

**Table 1:** Wealth Classification

Wealth classification	India				Chhattisgarh				Tamil Nadu			
	NFHS-4		NFHS-5		NFHS-4		NFHS-5		NFHS-4		NFHS-5	
	(2015-16)		(2019-21)		(2015-16)		(2019-21)		(2015-16)		(2019-21)	
	**N**	**%**	**N**	**%**	**N**	**%**	**N**	**%**	**N**	**%**	**N**	**%**
Poor	90,463	47	85,348	48	4,061	60	4,112	63	1,134	18	901	17
Non-poor	1,00,334	53	91,495	52	2,743	40	2,414	37	5,044	82	4,327	83

All statistical analyses were performed using Stata version 13.0 (Stata Corp LP, College Station, TX, USA).

### Ethics statement

Approval by the Ethics Committee is not necessary as this study is based on publicly available NFHS (secondary) dataset, containing no personally identifiable information.

## Results

### Section 1

**Fig. 1:**
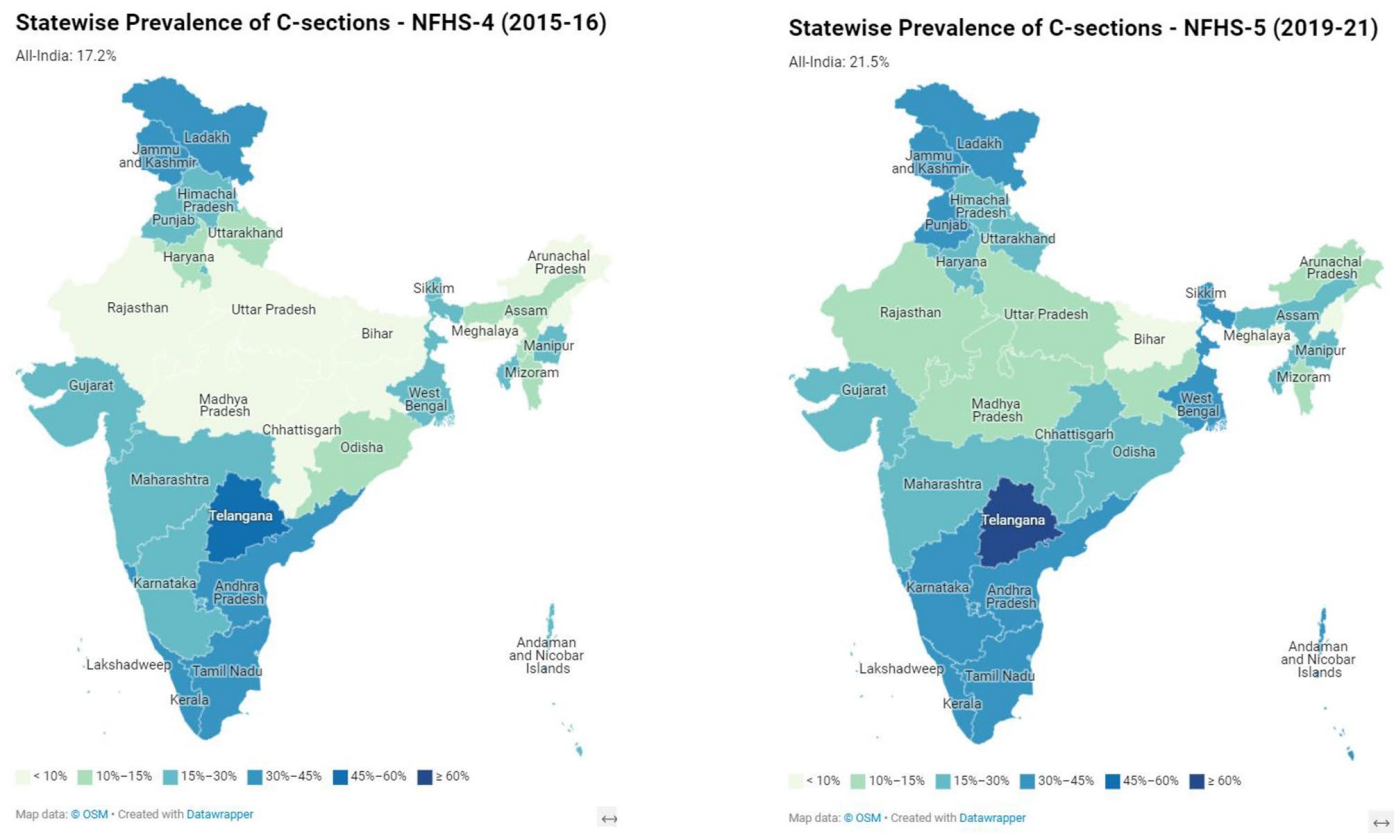
Prevalence of C-Section deliveries across the states of India (NFHS-4 and NFHS-5)

The percentage of caesarean deliveries across India increased from 17.2 to 21.5% between NFHS-4 (2015-16) and NFHS-5 (2019-21). All but four states and union territories in India experienced an increase in caesarean delivery rates over the 5 years studied. Earlier guidelines recommend that at the population level, 10 to 15% of all deliveries are C-sections; however, the WHO cautions there is no advantage in terms of reduction of infant or maternal mortality beyond a 10% population-wide C-section prevalence [33]. The proportion of C-sections at the all-India level had already crossed the WHO’s threshold in NFHS-4 (2015-16). C-section prevalence exceeded 15% in 22 out of 36 states and union territories in NFHS-4 (Fig. 1) and in 28 out of 36 in NFHS-5. Proportions of C-sections were lower than the recommended range in 3 states in NFHS-5 – in Nagaland (5.2%) and Meghalaya (8%), both high-focus North-eastern states, and in Bihar (9.7%).

### Respondents’ characteristics (Table 2)

Table 2 shows the characteristics of all those who gave birth to live children in India, CG and TN in the 5 years preceding 2015-16 and 2019-21 (NFHS-4 and − 5).

The majority of the respondents across India, TN and CG were Hindu (73.5% in India, 96.9% and in CG and 91.2% in TN) and belonged to Other Backward Castes (OBC) (CG is an exception where the majority – 43.8% - belonged to Scheduled Tribes) in NFHS-5. Across all regions, the majority resided in rural areas, were aged between 25 and 34, had received a secondary education, were married after they turned 18, and belonged to the non-poor category, except in CG where the majority (63% in NFHS-5) were poor. Close to a quarter of the respondents in CG were illiterate in both rounds of NFHS, while in TN only 5.6% and 1.6% were. The majority (58.7% in India, 61.2% in CG and 91.6% in TN in NFHS-5) said they had visited an antenatal care (ANC) facility 4 times or more.

Across regions, the majority of women had a normal BMI, but there is a marked difference in the proportion of overweight or obese women (1.6% in CG as compared to 12.9% in TN in NFHS-5). A majority of the respondents said they had given birth to average-sized babies. Most (60.5% in India, 66.8% in CG and 69.3% in TN) said they had no pregnancy complications in NFHS-5. A third of the respondents in India and CG and 40% in TN had given birth to their first child, and another 35% in India and in CG (47% in TN) to their second.

The distribution of respondents across wealth categories differed between the states, with 63% classified as “poor” in CG, while in TN only 17.2% were poor in NFHS-5. The proportion of women delivering in public facilities was far higher in CG (84.3%) when compared to that in TN (66.1%) in NFHS-5.

### Trends across NFHS-4 and NFHS-5 (Table 2)

In India, among all women who gave birth to a living child, women’s illiteracy decreased from 28.9 to 20.3%, with Secondary and Higher-level education seeing the highest gains. The number of ANC visits were also correspondingly higher, with the percentage of respondents who had never visited reducing from 18.6 to 6.5% and those who had visited more than 4 times increasing from 46.9 to 58.7%. However, the proportion of women who said they had no exposure to media increased in NFHS-5 across all the regions studied. Fewer (20% in NFHS-5 compared to 22.9% in NFHS-4) women were underweight. The percentage of women who were below 18 when they married decreased from 35.6 to 30.3%. Pregnancy complications were experienced by only 39.5% in NFHS-5 as compared to 42.2% in NFHS-4. Overall, public sector deliveries increased by 3% from 71.3 to 74.1%.

In Chhattisgarh, 7.8% more people lived in rural areas in 2019-21 as compared to 2015-16 (83.3% vs. 75.5%). Those who were younger than 18 when they married decreased by 7.1% (from 33.3 to 26.2%). 6% (8.7% in 2019-21 vs. 14.7% in 2015-16) fewer had had a pregnancy terminated in the past. The proportion of babies reported as “large” increased in CG (14.3% in NFHS-4 to 21.1% in NFHS-5). As in all-India, but to a more pronounced degree, pregnancy complications experienced were fewer (33% in 2019-21 vs. 44% in 2015-16). 5.1% more of all deliveries (from 79.2 to 84.3%) were in the public sector.

In Tamil Nadu, 59.6% people lived in rural areas in 2019-21 as compared to 56.5% in 2015-16. 17.2% more women had received higher education (40.3% in 2019-21 vs. 23.1% in 2015-16), and 91.6% had visited ANCs at least 4 times in 2019-21 compared to 80.8% in 2015-16. 84.9% were above 18 years of age at marriage in 2019-21 compared to 80.3% in 2015-16, and 5.3% more (12.9% in 2019-21 vs. 7.6% in 2015-16) women were overweight. The presence of pregnancy complications was lower in Tamil Nadu as well (30.7% in 2019-21 vs. 44.5% in 2015-16). Public sector deliveries decreased very slightly from 66.4 to 66.1%.

**Table 2:** Descriptive statistics of the study participants in India

Variable	India		Chhattisgarh		Tamil Nadu	
	NFHS-4	NFHS-5	NFHS-4	NFHS-5	NFHS-4	NFHS-5
	(2015-16)	(2019-21)	(2015-16)	(2019-21)	(2015-16)	(2019-21)
	**N**	**N**	**N**	**N**	**N**	**N**
	**(%)**	**(%)**	**(%)**	**(%)**	**(%)**	**(%)**
**Caste**						
Scheduled Castes	35,151	35,271	753	824	1,848	1,469
	(19.2)	(21.0)	(11.1)	(13.0)	(30.0)	(28.2)
Scheduled Tribes	37,856	35,379	2,858	2,779	111	91
	(20.7)	(21.1)	(42.1)	(43.8)	(1.8)	(1.7)
Other Backward Castes	74,033	67,024	2,683	2,523	4,111	3,572
	(40.5)	(39.9)	(39.5)	(39.8)	(66.8)	(68.4)
Others	35,869	30,373	495	220	89	87
	(19.6)	(18.1)	(7.3)	(3.5)	(1.5)	(1.7)
**Religion**						
Hindu	1,38,263	1,29,944	6,477	6,326	5,532	4,766
	(72.5)	(73.5)	(95.2)	(96.9)	(89.5)	(91.2)
Muslim	29,300	25,234	168	78	323	207
	(15.4)	(14.3)	(2.5)	(1.2)	(5.2)	(4.0)
Others	23,234	21,665	159	122	323	255
	(12.2)	(12.3)	(2.4)	(1.9)	(5.2)	(4.9)
**Type of Residence**						
Urban	47,814	37,975	1,669	1,088	2,685	2,110
	(25.1)	(21.5)	(24.5)	(16.7)	(43.5)	(40.4)
Rural	1,42,983	1,38,868	5,135	5,438	3,493	3,118
	(74.9)	(78.5)	(75.5)	(83.3)	(56.5)	(59.6)
**Woman’s education level**						
Illiterate	55,105	35,976	1,720	1,528	343	84
	(28.9)	(20.3)	(25.3)	(23.4)	(5.6)	(1.6)
Primary	26,696	21,737	1,327	853	430	240
	(14.0)	(12.3)	(19.5)	(13.1)	(7.0)	(4.6)
Secondary	88,847	92,624	3,191	3,519	3,976	2,797
	(46.6)	(52.4)	(46.9)	(53.9)	(64.4)	(53.5)
Higher	20,149	26,506	566	626	1,429	2,107
	(10.6)	(15.0)	(8.3)	(9.6)	(23.1)	(40.3)
**Age at marriage**						
Below 18 Years	66,368	53,629	2,223	1,710	1,200	791
	(35.6)	(30.3)	(33.3)	(26.2)	(19.7)	(15.1)
18 Years or Above	1,21,174	1,23,214	4,459	4,808	4,878	4,436
	(64.6)	(69.7)	(66.7)	(73.8)	(80.3)	(84.9)
**Exposure to media**						
Nil	49,336	48,997	1,359	1,965	114	204
	(25.9)	(27.7)	(20.0)	(30.1)	(1.9)	(3.9)
Partial	1,26,851	1,15,724	5,079	4,202	5,019	4,536
	(66.5)	(65.4)	(74.7)	(64.4)	(81.2)	(86.8)
Full	14,610	12,122	366	359	1,045	488
	(7.7)	(6.9)	(5.4)	(5.5)	(16.9)	(9.3)
**Number of ANC visits**						
0	35,395	11,462	305	339	566	185
	(18.6)	(6.5)	(4.5)	(5.2)	(9.2)	(3.5)
1–3	65,964	61,586	2,630	2,191	621	254
	(34.6)	(34.8)	(38.7)	(33.6)	(10.1)	(4.9)
4+	89,438	1,03,795	3,869	3,996	4,991	4,789
	(46.9)	(58.7)	(56.9)	(61.2)	(80.8)	(91.6)
**Woman’s age**						
15–24	62,079	53,635	2,264	1,847	1,996	1,467
	(32.5)	(30.3)	(33.3)	(28.3)	(32.3)	(28.1)
25–34	1,07,471	1,04,032	3,934	4,041	3,828	3,339
	(56.3)	(58.8)	(57.8)	(61.9)	(62.0)	(63.9)
35–49	21,247	19,176	606	638	354	422
	(11.1)	(10.9)	(8.9)	(9.8)	(5.7)	(8.1)
**Size of child at birth**						
Small	22,722	17,696	628	665	574	471
	(12.2)	(10.1)	(9.4)	(10.2)	(9.3)	(9.0)
Average	1,30,586	1,24,392	5,107	4,465	4,008	3,899
	(69.9)	(71.2)	(76.3)	(68.6)	(65.0)	(74.6)
Large	33,476	32,619	957	1,375	1,582	855
	(17.9)	(18.7)	(14.3)	(21.1)	(25.7)	(16.4)
**Woman’s BMI**						
Underweight	43,161	35,426	1,789	1,651	871	609
	(22.9)	(20.0)	(26.5)	(25.9)	(14.3)	(11.9)
Normal	1,39,472	1,08,342	4,859	4,624	4,775	3,843
	(74.1)	(61.3)	(71.9)	(72.5)	(78.2)	(75.2)
Overweight	5,569	33,075	107	101	464	661
	(3.0)	(18.7)	(1.6)	(1.6)	(7.6)	(12.9)
**Pregnancy complications**						
No	1,10,217	1,07,010	3,827	4,358	3,429	3,623
	(57.8)	(60.5)	(56.3)	(66.8)	(55.5)	(69.3)
Present	80,580	69,833	2,977	2,168	2,749	1,605
	(42.2)	(39.5)	(43.8)	(33.2)	(44.5)	(30.7)
**Ever had a terminated pregnancy**						
No	1,60,970	1,50,580	5,801	5,957	5,304	4,335
	(84.4)	(85.2)	(85.3)	(91.3)	(85.9)	(82.9)
Yes	29,827	26,263	1,003	569	874	893
	(15.6)	(14.9)	(14.7)	(8.7)	(14.2)	(17.1)
**Birth Order**						
1	61,803	59,620	2,184	2,156	2,496	2,112
	(32.4)	(33.7)	(32.1)	(33.0)	(40.4)	(40.4)
2	62,468	62,370	2,374	2,280	2,823	2,451
	(32.7)	(35.3)	(34.9)	(34.9)	(45.7)	(46.9)
3	33,041	29,883	1,263	1,201	709	576
	(17.3)	(16.9)	(18.6)	(18.4)	(11.5)	(11.0)
4+	33,485	24,970	983	889	150	89
	(17.6)	(14.1)	(14.5)	(13.6)	(2.4)	(1.7)
**Wealth quintile**						
Poor	90,463	85,348	4,061	4,112	1,134	901
	(47.4)	(48.3)	(59.7)	(63.0)	(18.4)	(17.2)
Non-poor	1,00,334	91,495	2,743	2,414	5,044	4,327
	(52.6)	(51.7)	(40.3)	(37.0)	(81.7)	(82.8)
**Place of delivery**						
Public	1,05,615	1,14,952	3,905	4,684	4,069	3,445
	(71.3)	(74.1)	(79.2)	(84.3)	(66.4)	(66.1)
Private	42,570	40,280	1,023	874	2,061	1,766
	(28.7)	(26.0)	(20.8)	(15.7)	(33.6)	(33.9)

### Section 2

#### Prevalence of C-sections by selected explanatory variables (Tables 3, 4 and 5)

C-sections became more prevalent in both urban and in rural areas between 2015 and 16 and 2019-21 across all the regions studied. Prevalence has increased by 11.3% and 4.2% (54% and 50% relative) in CG in urban and rural areas respectively. The proportions of C-sections in TN are much higher, however, at 49.3% and 45.8% in NFHS-5 in urban and rural areas, while in CG, they are 32.2% and 12.6% respectively.

The prevalence of C-sections was highest among those who listed their religion under “others” in India and in TN and among Muslims in CG in both the rounds of NFHS compared. C-sections are most prevalent among those listing their castes as “others” across regions and NFHS rounds. The difference in proportion between the caste classifications is not so stark in TN (except for the Scheduled Tribes, where 34% of births are C-sections) when compared to that in CG (8.2% for Scheduled Tribes and 41.6% for Other Castes in NFHS-5) and India (12.8% for Scheduled Tribes and 31.1% for Other Castes).

A considerably higher proportion of women who had completed higher education had undergone C-sections in India (42.2%) and CG (42.5%) as compared to those who were illiterate (9.1% and 4.5% respectively) in NFHS-5, although this distinction was not so pronounced in TN (52.7% vs. 32.1% respectively). Similarly, the greater the exposure to media, the higher the prevalence of C-sections in India (34.5 for “full” and 11.2% for “none”) and in CG (28% and 7.6% respectively) in NFHS-5, but the prevalence was uniformly high in TN (50.1% vs. 48.8%).

At the all-India level, those who visited ANCs more than 4 times had nearly double the rates of C-Sect. (29.2% in NFHS-5) as those who did not visit at all (15.9%). In CG and TN, however, the prevalence of C-sections only varied by 4.9% and 3.5% respectively depending on the number of ANC visits. In India and in CG, the prevalence of C-sections doubled among women who married when they were 18 or older (27.9% in India and 19.4% in CG) when compared with those who married when younger than 18 (16% in India and 8.8% in CG), while in Tamil Nadu, it was at 38% and 49% respectively in NFHS-5.

The prevalence of C-sections increased with age in NFHS-5. Over the 5-year period in between the rounds compared, in TN, C-sections increased relatively by 33% (from 30.4 to 40.3%), 30% (from 37.6 to 48.8%) and 32% (from 45.8 to 60.3%) respectively for the 15–24, 25–34 and the 35–49 age groups. In CG, the highest absolute proportion was 17.8% for women aged 35–49 in NFHS-5.

As BMI increased, so did the proportion of women who delivered via C-sections in India, TN and CG. The proportion of C-sections was highest for children whose size at the time of birth was reported as “large” in India (26.4%), as “average” in CG (17.4%), and as “small” in TN (50.5%). Across regions, a higher percentage of women who had a terminated pregnancy had C-sections than those who did not have a terminated pregnancy (+ 4.3% all-India, + 3.6% Chhattisgarh and + 3.7% Tamil Nadu), as did those who had pregnancy complications compared to those who did not (+ 4% all-India, + 5.9% Chhattisgarh and + 6.1% Tamil Nadu). An inverse relationship was observed between the prevalence of C-sections and birth order of the child.

Interestingly, across regions, almost half or more of deliveries in the private sector (49.7% in India, 58.9% in CG, 64.2% in TN) were C-sections, much higher than the 16.1%, 9.7% and 38.9% in the public sector India, CG and TN respectively in NFHS-5.

The prevalence of C-sections increased with wealth, although not as starkly in TN (where rates are at 38.9% even for the poor) as in India and in CG.

**Table 3:** Prevalence of Caesarean section delivery by selected explanatory variables at the all-India level

India	NFHS-4		NFHS-5	
	(2015-16)		(2019-21)	
Variable	%	p-value	%	p-value
**Caste**		< 0.001		< 0.001
Scheduled Castes	16.4		21.2	
Scheduled Tribes	9.6		12.8	
Other Backward Castes	19.2		24.5	
Others	25.6		31.1	
**Religion**		< 0.001		< 0.001
Hindu	19.4		24.0	
Muslims	17.0		21.9	
Others	23.8		31.4	
**Type of residence**		< 0.001		< 0.001
Urban	30.4		34.7	
Rural	14.5		19.8	
**Woman’s education level**		< 0.001		< 0.001
Illiterate	6.9		9.1	
Primary	12.6		14.5	
Secondary	23.1		25.7	
Higher	39.8		42.2	
**Age at marriage**		< 0.001		< 0.001
Below 18 years	12.3		16.0	
18 years or above	23.4		27.9	
**Exposure to media**		< 0.001		< 0.001
Nil	5.8		11.2	
Partial	22.8		28.1	
Full	30.6		34.5	
**Number of ANC visits**		< 0.001		< 0.001
0	7.1		15.9	
1–3	12.1		16.8	
4+	27.7		29.2	
**Woman’s age**		< 0.001		< 0.001
15–24	18.3		21.1	
25–34	20.2		25.3	
35–49	16.9		25.7	
**Size of child at birth**		< 0.001		< 0.001
Average	18.4		23.5	
Small	17.1		24.4	
Large	24.2		26.4	
**Woman’s BMI**		< 0.001		< 0.001
Underweight	11.1		15.1	
Normal	20.3		24.3	
Overweight	47.1		50.0	
**Pregnancy Complications**		< 0.001		< 0.001
No	18.3		22.4	
Present	20.5		26.4	
**Ever had a terminated pregnancy**		< 0.001		< 0.001
No	18.7		23.3	
Yes	22.3		27.6	
**Birth order**		< 0.001		< 0.001
1	27.5		32.0	
2	21.9		27.0	
3	11.0		14.4	
4+	4.2		6.5	
**Wealth quintile**		< 0.001		< 0.001
Poor	7.9		12.7	
Non-poor	28.3		32.8	
**Place of delivery**		< 0.001		< 0.001
Public	13.2		16.1	
Private	43.1		49.7	

**Table 4:** Prevalence of Caesarean section delivery by selected explanatory variables in Chhattisgarh

Chhattisgarh	NFHS-4		NFHS-5	
	(2015-16)		(2019-21)	
Variable	%	p-value	%	p-value
**Caste**		0.015		< 0.001
Scheduled Castes	10.4		16.1	
Scheduled Tribes	4.8		8.2	
Other Backward Castes	12.8		20.3	
Others	31.7		41.6	
**Religion**		< 0.001		0.026
Hindu	10.7		16.5	
Muslim	27.9		31.8	
Others	13.6		20.6	
**Type of Residence**		< 0.001		< 0.001
Urban	20.9		32.2	
Rural	8.4		12.6	
**Woman’s education level**		< 0.001		< 0.001
Illiterate	4.2		4.5	
Primary	7.3		9.4	
Secondary	12.0		16.6	
Higher	33.7		42.5	
**Age at marriage**		< 0.001		< 0.001
Below 18 years	7.1		8.8	
18 years or above	13.3		19.4	
**Exposure to media**		< 0.001		< 0.001
Nil	4.0		7.6	
Partial	11.9		18.6	
Full	20.7		28.0	
**Number of ANC visits**		< 0.001		0.058
0	5.2		13.0	
1–3	7.4		15.4	
4+	13.9		17.9	
**Woman’s age**		0.015		0.117
15–24	9.5		14.2	
25–34	12.2		17.7	
35–49	10.9		17.8	
**Size of child at birth**		0.004		0.094
Average	10.2		17.4	
Small	14.5		14.4	
Large	14.8		15.5	
**Woman’s BMI**		< 0.001		< 0.001
Underweight	7.0		10.0	
Normal	11.9		18.0	
Overweight	40.2		43.4	
**Pregnancy Complications**		0.005		< 0.001
No	10.4		14.8	
Present	12.1		20.7	
**Ever had a terminated pregnancy**		< 0.001		0.005
No	10.5		16.4	
Yes	15.1		20.0	
**Birth Order**		< 0.001		< 0.001
1	16.4		22.3	
2	12.6		19.5	
3	5.7		8.0	
4+	3.0		5.4	
**Wealth Quintile**		< 0.001		< 0.001
Poor	5.1		8.4	
Non-poor	18.9		26.9	
**Place Of Delivery**		< 0.001		< 0.001
Public	6.3		9.7	
Private	48.5		58.9	

**Table 5:** Prevalence of Caesarean section delivery by selected explanatory variables in Tamil Nadu

Tamil Nadu	NFHS-4		NFHS-5	
	(2015-16)		(2019-21)	
Variable	%	p-value	%	p-value
**Caste**		< 0.001		< 0.001
Scheduled Castes	31.8		44.4	
Scheduled Tribes	30.8		34.0	
Other Backward Castes	37.4		48.9	
Others	46.1		49.6	
**Religion**		< 0.001		0.026
Hindu	35.0		47.3	
Muslim	39.3		45.4	
Others	46.1		50.3	
**Type of Residence**		0.001		0.008
Urban	37.5		49.3	
Rural	34.2		45.8	
**Woman’s education level**		< 0.001		< 0.001
Illiterate	26.8		32.1	
Primary	31.3		46.9	
Secondary	34.5		43.8	
Higher	42.5		52.7	
**Age at marriage**		< 0.001		< 0.001
Below 18 years	27.2		38.1	
18 years or above	37.8		49.0	
**Exposure to media**		0.478		0.706
Nil	32.9		48.8	
Partial	36.2		47.0	
Full	34.1		50.1	
**Number of ANC visits**		0.032		0.058
0	32.3		46.5	
1–3	31.7		50.0	
4+	36.7		47.3	
**Woman’s age**		< 0.001		0.117
15–24	30.4		40.3	
25–34	37.6		48.8	
35–49	45.8		60.3	
**Size of child at birth**		0.082		0.011
Average	34.6		46.5	
Small	35.8		50.5	
Large	38.7		49.7	
**Woman’s BMI**		< 0.001		< 0.001
Underweight	25.9		33.8	
Normal	35.4		46.6	
Overweight	57.3		60.6	
**Pregnancy Complications**		0.197		< 0.001
No	34.7		45.5	
Present	42.6		51.6	
**Ever had a terminated pregnancy**		< 0.001		0.054
No	34.8		46.8	
Yes	37.1		50.5	
**Birth Order**		< 0.001		< 0.001
1	39.2		52.4	
2	36.8		47.0	
3	23.4		32.7	
4+	15.1		28.1	
**Wealth Quintile**		< 0.001		< 0.001
Poor	28.6		38.9	
Non-poor	37.3		48.8	
**Place Of Delivery**		< 0.001		< 0.001
Public	27.6		38.9	
Private	52.7		64.2	

### Section 3

#### Factors associated with caesarean section delivery (Table 6)

Table 6 presents the results of the multivariate logistic regression models assessing the factors associated with caesarean section delivery for India and for Chhattisgarh and Tamil Nadu. The odds of most of the explanatory variables remained similar across both the rounds of NFHS (4 and 5) compared.

Women’s age at marriage did not significantly affect the likelihood of C-sections. However, those who had completed a higher education were 1.2 times more likely in India and 1.9 times more likely in Chhattisgarh to have delivered via a C-section. In Tamil Nadu, the impact of education was not significant. In India, 4 or more ANC visits meant 1.2 times higher odds of C-sections. Across India and in TN, the likelihood of the delivery being via C-section decreased with increasing birth order.

The odds of having a C-section increased with the age of the mother and are 2.1 times for women in the 35–49 bracket when compared to those aged 15–24. Women who reported the size of the child as smaller or larger than average were more likely to have undergone C-sections. The presence of pregnancy complications increased the likelihood of C-sections in CG and in TN by 1.5 and 1.2 times respectively. While the presence of a previously terminated pregnancy meant a 1.2 times higher likelihood of C-sections, women’s BMI was a significant factor influencing the outcome – women who were overweight were between 2.1 times more likely than those who were underweight to have had a C-section in NFHS-5.

The non-poor were more likely than the poor to have a C-section in India and in Chhattisgarh. However, the results were inconclusive for Tamil Nadu. The most significant factor we observed was the place of delivery (private or public facility). The odds of having a C-section were much higher in a private facility when compared to a public facility – 3.9 times so in India, 2.7 times in Tamil Nadu and an astounding 9.6 times in Chhattisgarh in NFHS-5.

**Table 6:** Multivariate logistic regression models assessing the factors associated with the caesarean section delivery in India, Tamil Nadu, and Chhattisgarh (NFHS-4; 2015-16 & NFHS-5;2019-21)

Factor	India		Chhattisgarh		Tamil Nadu	
	NFHS-4	NFHS-5	NFHS-4	NFHS-5	NFHS-4	NFHS-5
	(2015-16)	(2019-21)	(2015-16)	(2019-21)	(2015-16)	(2019-21)
**Caste**						
Scheduled Castes	1[Reference]	1[Reference]	1[Reference]	1[Reference]	1[Reference]	1[Reference]
Scheduled Tribes	0.76***	0.68***	0.70*	0.69*	1.19	0.58*
	(0.69–0.84)	(0.63–0.74)	(0.48–1.02)	(0.47–1.01)	(0.69–2.05)	(0.33–1.04)
Other Backward Castes	0.89***	0.90***	0.96	1.00	0.97	0.93
	(0.84–0.95)	(0.85–0.95)	(0.68–1.35)	(0.73–1.38)	(0.83–1.14)	(0.77–1.13)
Others	0.93*	0.94*	1.62**	1.11	1.31	0.88
	(0.87–1.01)	(0.88–1.01)	(1.05–2.52)	(0.65–1.91)	(0.86–2.00)	(0.50–1.53)
**Religion**						
Hindu	1[Reference]	1[Reference]	1[Reference]	1[Reference]	1[Reference]	1[Reference]
Muslim	0.95	0.96	1.29	1.09	1.04	0.87
	(0.88–1.02)	(0.89–1.02)	(0.64–2.62)	(0.39–3.01)	(0.73–1.47)	(0.61–1.22)
Others	0.98	1.16***	0.52*	0.39**	1.26	0.98
	(0.89–1.09)	(1.06–1.28)	(0.25–1.08)	(0.17–0.87)	(0.94–1.69)	(0.68–1.39)
**Type of Residence**						
Urban	1[Reference]	1[Reference]	1[Reference]	1[Reference]	1[Reference]	1[Reference]
Rural	0.86***	0.89***	1.24	0.78	1.16*	1.18**
	(0.81–0.91)	(0.85–0.94)	(0.94–1.63)	(0.57–1.07)	(0.99–1.35)	(1.02–1.37)
**Woman’s education level**						
Illiterate	1[Reference]	1[Reference]	1[Reference]	1[Reference]	1[Reference]	1[Reference]
Primary	1.20***	1.15***	1.24	1.38	1.16	1.60*
	(1.09–1.32)	(1.06–1.26)	(0.79–1.94)	(0.82–2.31)	(0.78–1.72)	(0.92–2.78)
Secondary	1.24***	1.40***	1.09	1.47*	1.13	1.29
	(1.16–1.34)	(1.31–1.50)	(0.70–1.70)	(0.96–2.25)	(0.83–1.54)	(0.77–2.14)
Higher	1.25***	1.21***	1.09	1.94**	1.01	1.12
	(1.14–1.37)	(1.15–1.27)	(0.64–1.86)	(1.15–3.25)	(0.71–1.44)	(0.66–1.90)
**Age at marriage**						
Below 18 years	1[Reference]	1[Reference]	1[Reference]	1[Reference]	1[Reference]	1[Reference]
18 Years or above	1.05*	1	0.8	1.02	1.15	1.14
	(1.00–1.11)	(0.95–1.06)	(0.59–1.10)	(0.72–1.43)	(0.96–1.38)	(0.91–1.42)
**Exposure to media**						
Nil	1[Reference]	1[Reference]	1[Reference]	1[Reference]	1[Reference]	1[Reference]
Partial	1.40***	1.34	1.34***	1.13	0.99	0.77
	(1.30–1.50)	(1.26–1.42)	(1.26–1.42)	(0.82–1.56)	(0.58–1.68)	(0.55–1.07)
Full	1.43***	1.33	1.33***	1.28	0.81	0.71*
	(1.28–1.60)	(1.21–1.46)	(1.21–1.46)	(0.80–2.05)	(0.46–1.42)	(0.48–1.05)
**Number of ANC Visits**						
0	1[Reference]	1[Reference]	1[Reference]	1[Reference]	1[Reference]	1[Reference]
1–3	1.13***	0.88***	0.84	1.05	0.95	1.33
	(1.03–1.23)	(0.80–0.97)	(0.37–1.90)	(0.62–1.78)	(0.67–1.33)	(0.82–2.14)
4+	1.72***	1.23***	1.13	0.94	1.17	1.21
	(1.58–1.87)	(1.12–1.35)	(0.51–2.51)	(0.56–1.58)	(0.91–1.49)	(0.87–1.69)
**Woman’s age**						
15–24	1[Reference]	1[Reference]	1[Reference]	1[Reference]	1[Reference]	1[Reference]
25–34	1.20***	1.36***	1.32**	1.26*	1.31***	1.37***
	(1.14–1.26)	(1.30–1.43)	(1.01–1.72)	(0.96–1.64)	(1.11–1.54)	(1.14–1.64)
35–49	1.74***	2.10***	1.66*	2.15***	2.00***	2.22***
	(1.57–1.92)	(1.92–2.29)	(0.97–2.84)	(1.37–3.39)	(1.40–2.87)	(1.63–3.02)
**Size of child at birth**						
Average	1[Reference]	1[Reference]	1[Reference]	1[Reference]	1[Reference]	1[Reference]
Small	1.10***	1.17***	1.42*	0.70*	1.07	1.31**
	(1.03–1.18)	(1.10–1.25)	(0.96–2.09)	(0.48–1.02)	(0.84–1.35)	(1.02–1.68)
Large	1.31***	1.20***	1.44**	0.89	1.16**	1.11
	(1.24–1.39)	(1.14–1.26)	(1.08–1.92)	(0.65–1.20)	(1.00–1.34)	(0.92–1.34)
**Woman’s BMI**						
Underweight	1[Reference]	1[Reference]	1[Reference]	1[Reference]	1[Reference]	1[Reference]
Normal	1.37***	0.75***	1.23	0.79	1.33***	0.70***
	(1.29–1.45)	(0.70–0.79)	(0.92–1.66)	(0.59–1.05)	(1.10–1.61)	(0.56–0.87)
Overweight	2.91***	2.11***	2.53**	2.13***	2.85***	1.69***
	(2.54–3.34)	(1.95–2.29)	(1.19–5.39)	(1.20–3.76)	(2.06–3.93)	(1.37–2.07)
**Pregnancy complications**						
No	1[Reference]	1[Reference]	1[Reference]	1[Reference]	1[Reference]	1[Reference]
Present	1.14***	1.15	1.21	1.52***	1.08	1.20**
	(1.09–1.19)	(1.10–1.20)	(0.96–1.52)	(1.22–1.89)	(0.93–1.24)	(1.03–1.39)
**Ever had a terminated pregnancy**						
No	1[Reference]	1[Reference]	1[Reference]	1[Reference]	1[Reference]	1[Reference]
Yes	1.20***	1.18***	1.16	1.01	0.94	1.14
	(1.12–1.27)	(1.12–1.24)	(0.88–1.53)	(0.71–1.44)	(0.78–1.13)	(0.95–1.36)
**Birth order**						
1	1[Reference]	1[Reference]	1[Reference]	1[Reference]	1[Reference]	1[Reference]
2	0.81***	0.80***	0.82	1.01	0.91	0.76***
	(0.76–0.85)	(0.76–0.84)	(0.63–1.07)	(0.78–1.29)	(0.78–1.06)	(0.65–0.88)
3	0.47***	0.42***	0.49***	0.48***	0.46***	0.37***
	(0.44–0.51)	(0.39–0.45)	(0.31–0.79)	(0.33–0.72)	(0.36–0.59)	(0.29–0.49)
4 or more	0.25***	0.23***	0.35***	0.40***	0.24***	0.33***
	(0.22–0.28)	(0.20–0.25)	(0.20–0.63)	(0.24–0.66)	(0.14–0.41)	(0.18–0.59)
**Wealth quintile**						
Poor	1[Reference]	1[Reference]	1[Reference]	1[Reference]	1[Reference]	1[Reference]
Non-poor	1.32***	1.21***	1.43**	1.2	0.94	1.02
	(1.24–1.40)	(1.15–1.27)	(1.08–1.89)	(0.91–1.57)	(0.78–1.13)	(0.85–1.24)
**Place of delivery**						
Public	1[Reference]	1[Reference]	1[Reference]	1[Reference]	1[Reference]	1[Reference]
Private	3.65***	3.90***	10.02***	9.57***	2.64***	2.65***
	(3.46–3.84)	(3.74–4.06)	(7.63–13.16)	(7.51–12.20)	(2.25–3.09)	(2.27–3.10)
Constant	0.07***	0.08***	0.06***	0.06***	0.33***	0.42***
	(0.06–0.08)	(0.07–0.09)	(0.02–0.16)	(0.03–0.14)	(0.16–0.68)	(0.19–0.87)
Observations	1,36,814	1,42,661	4,757	5,257	5,939	5,083

95% CI values in parentheses; *** p < 0.01, ** p < 0.05, * p < 0.1

### Section 4

Given that the place of delivery (private or public facility) is the most significant factor influencing whether delivery was by C-sections, this section further analyses the differences in prevalence.

**Fig. 2:**
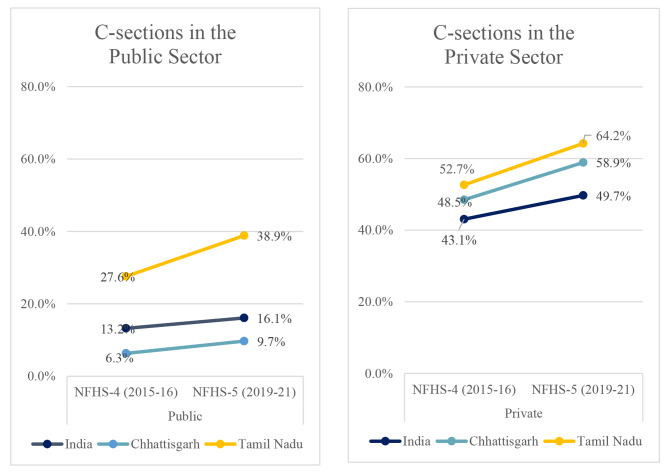
Proportions of C-sections in the Public and Private Sectors for NFHS-3, 4 and 5

In public sector hospitals, the proportion of C-section deliveries increased from 13.2 to 16.1% in India between 2015 and 16 and 2019-21 (Fig. 2). It increased particularly significantly in Tamil Nadu from 27.6 to 38.9% across the same period. In Chhattisgarh, the proportion increased from 6.3% in 2015-16 to 9.7% in 2019-21. In private institutions, the proportions of C-sections are uniformly higher than those in the public sector and have increased over the years in question across India, Tamil Nadu, and Chhattisgarh (Fig. 2).

### Inequality in public facilities

Analysing for wealth-related inequality in proportions of C-sections in public institutions shows that absolute inequality was much higher in India and in CG than in TN (Table 7).

Both absolute and relative inequality increased in CG, and both, already at comparatively low levels, decreased in TN, implying that all wealth quintiles undergo C-sections similar rates in the public sector in TN. In India absolute inequality rose, but relative inequality decreased.

**Table 7:** Slope Index of inequality (SII) and Relative concentration Index (CIX) for caesarean sections in public facilities, NFHS-4 & 5

**SII**				
**Region**	**SII**	**95% CI**	**SII**	**95% CI**
	**NFHS-4**		**NFHS-5**	
	**(2015-16)**		**(2019-21)**	
India	20.3*	(19.7–20.9)	22.9*	(22.3–23.6)
CG	7.9*	(5.3–10.4)	14*	(11.3–16.7)
TN	4.7*	(0.4-9.0)	4.6**	(-0.6-9.7)
**p < 0.05 *p < 0.001				
**CIX**				
**Region**	**CIX**	**95% CI**	**CIX**	**95% CI**
	**NFHS-4**		**NFHS-5**	
	**(2015-16)**		**(2019-21)**	
India	29.2*	(28.4–29.9)	28.5*	(27.8–29.2)
CG	21.5*	(14.8–28.2)	26.4*	(21.0-31.9)
TN	2.94**	(0.4–5.5)	2.2*	(-0.01-4.5)

**p < 0.05 *p < 0.001

**Fig. 3:**
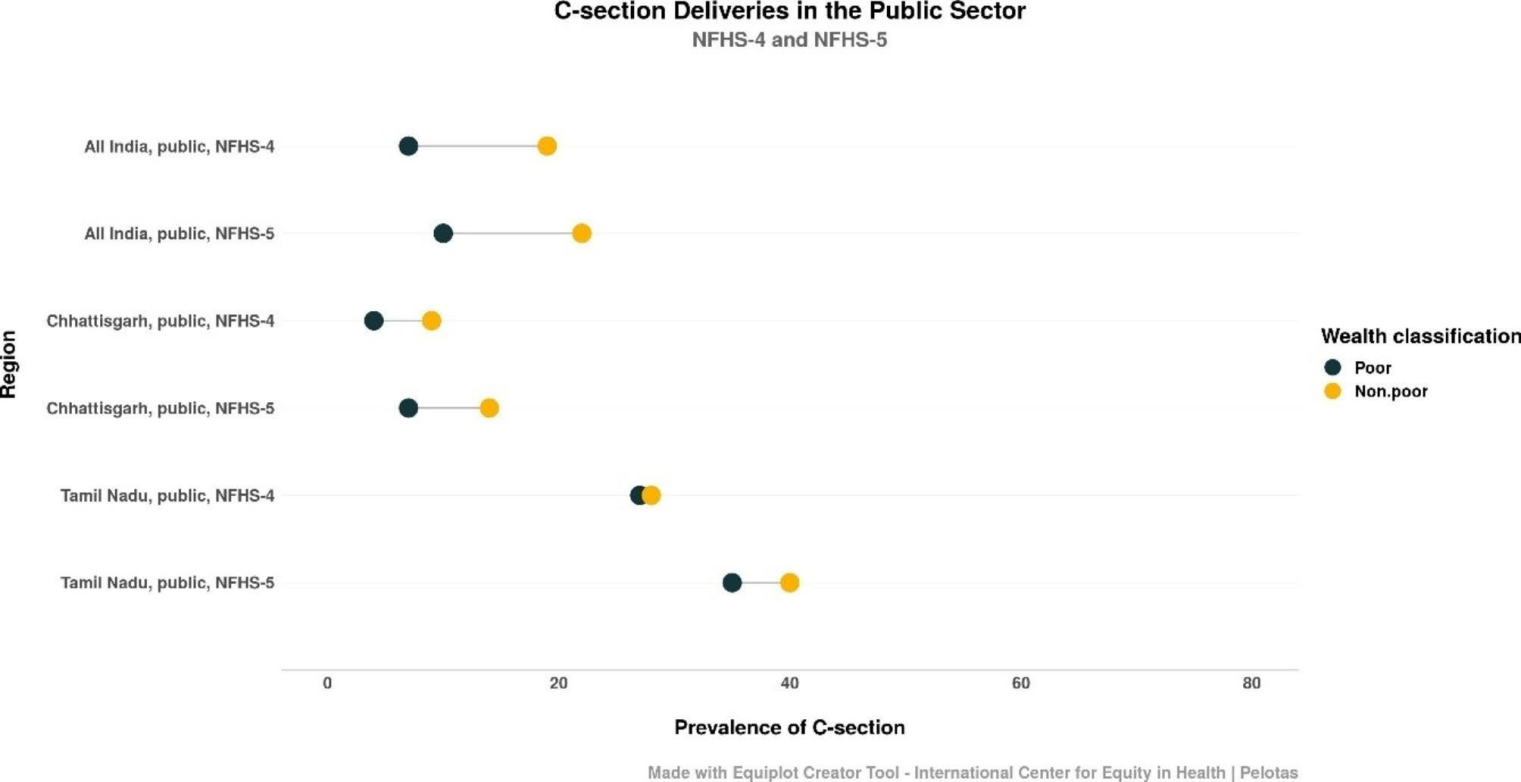
Trends in C-section prevalence for the poor and the non-poor in public facilities in India, TN and CG across NFHS-4 and − 5

Figure 3 is an equiplot that shows the trends in C-section prevalence in public facilities where the lower two quintiles have been classified as “poor” and the upper three as “non-poor”. This indicates that women from non-poor households have a higher proportion of C-sections in public health facilities when compared to their poor counterparts across regions and NFHS rounds. The equiplot shows both prevalence and inequality rising across India, Chhattisgarh and Tamil Nadu.

### Inequality in private facilities

In private institutions, both absolute and relative inequality across India decreased over the years between the rounds (Table 8). A higher proportion of women from all households underwent C-sections in private facilities in 2019-21, while the gap between the non-poor and the poor decreased, implying that poor women increasingly delivered via C-sections in private facilities. In CG, relative inequality increased. In TN, surprisingly, a negative absolute inequality measure indicated that the poorest quintile had C-section rates that were 7.6% higher than the richest quintile.

**Table 8:** Slope Index of inequality (SII) and Relative concentration Index (CIX) for caesarean sections in private facilities, NFHS-4 & 5

**SII**				
**Region**	**SII**	**95% CI**	**SII**	**95% CI**
	**NFHS-4**		**NFHS-5**	
	**(2015-16)**		**(2019-21)**	
India	19.6 *	(18.2–21.0)	14.3*	(12.7–15.8)
CG	8.2	(-1.7-18.2)	14.4*	(3.85–24.9)
TN	-4.3	(-11.5-2.8)	-7.6**	(-15.0 - -0.1)
**p < 0.05 *p < 0.001				
**CIX**				
**Region**	**CIX**	**95% CI**	**CIX**	**95% CI**
	**NFHS-4**		**NFHS-5**	
	**(2015-16)**		**(2019-21)**	
India	8.5*	(7.8–9.1)	6.6*	(6.1–7.1)
CG	3.2***	(-0.2-6.6)	4.1*	(0.9–7.3)
TN	-1.35	(-3.5-0.8)	-0.95	(-2.8-0.9)

***p < 0.100 **p < 0.05 *p < 0.001

**Fig. 4:**
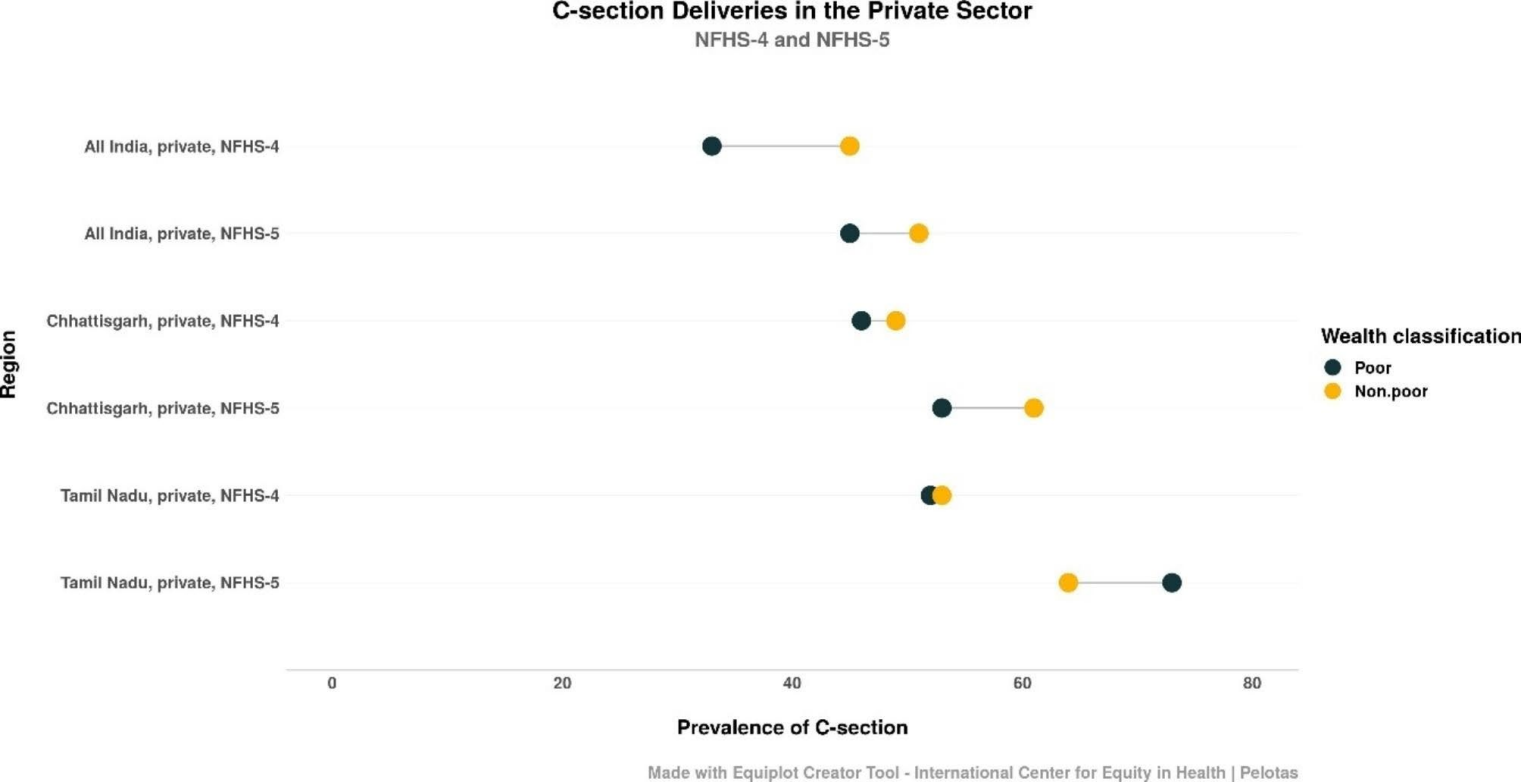
Trends in C-section prevalence for the poor and the non-poor in private facilities in India, TN and CG across NFHS-4 and 5

Figure 4 plots the trends in C-section prevalence in private facilities where the lower two quintiles have been classified as “poor” and the upper three as “non-poor”. Prevalence of C-sections increased for both wealth categories and the gap increased as well in private facilities in 2019-21 in Chhattisgarh. In Tamil Nadu, as with the absolute inequality index, the equiplot shows that a higher proportion of women from poor households underwent C-sections in private health facilities as compared to their non-poor counterparts in NFHS-5.

## Discussion

28 out of 36 states and union territories in India exceeded the 15% upper limit of the “ideal rate” for C-sections. This threshold might be too low; analysis of data from all 194 WHO member-states in 2012 pegs the optimal caesarean delivery rate at 19% [34]. Earlier research that suggested thresholds ideal for maternal and newborn mortality were arguably lower than optimum because they looked at data from a small number of countries, frequently focussed on results in wealthier nations and did not consider morbidity in addition to mortality [35–37]. A lower prevalence of C-section is not necessarily an end in itself – in Haiti, for example, overall rates are at 5.4% but there are considerable geographic, economic and social inequalities [14]. Specialists with whom the authors had a discussion on thresholds for C-sections also opined that threshold levels, if they are to be used at all, need a relook and should be revised upwards. They are of the view that it could be placed at around 30% - while they did not consider this as desirable, they viewed this revision as medically necessary given the poor health (nutritional) status of women, particularly in India.

Notwithstanding these considerations, while some of the rise in C-section prevalence may be attributed to medical or allied reasons such as increasing obesity and older ages of the mother at delivery, the prevalence of pregnancy complications appears to be decreasing in the overall study sample, indicating that other non-medical reasons influence mode of delivery. A recent study found that 21% of C-section deliveries in India, and more than 40% of those in private hospitals and in the south, were performed on women whose pregnancies were classified as low risk [38]. Economic incentives, women’s own preferences [18, 19], their socio-economic level and education, and risk-averse physicians practising conservative medicine could be some of these non-clinical factors [39–43].

Women residing in urban regions had a higher likelihood of delivering via C-section than their rural counterparts. This could be due to the availability and accessibility of advanced treatment and private health facilities, and because women are better educated and have higher autonomy in urban areas [44–46].

In line with findings from previous studies conducted in South-East Asian regions [7, 39, 45], our analysis showed that maternal age is significantly associated with caesarean deliveries. The likelihood of delivery by C-section increases with maternal age across the world [47], as older women are more likely to suffer from obstetric complications [48]. C-section deliveries are more prevalent among women belonging to the “general” *caste* in 2019-21. Compared to the general and the backward *castes*, scheduled *castes* and scheduled tribes still live in slums and in segregated villages, according to a recent study conducted in Bihar [49]. Use of maternal health services by these groups, therefore, also remains comparatively lower [49, 50], possibly leading to a lower prevalence of C-sections.

We observed an inverse association between birth order of the child and C-section deliveries – some studies suggest that as mothers gain more experience, they become more aware of when to seek care in order to avert complications and therefore do not need C-Sects [7, 44]. Increasing exposure to media was not associated with higher odds of C-sections in 2019-21, contrary to results in previous studies [10].

The number of ANC visits is on the rise across the country, as is women’s literacy, leading to greater opportunities for patient education on the adverse effects of unnecessary C-sections. Both these factors are positively associated with C-section prevalence, however. One reason could be that the emphasis that healthcare staff place on safe deliveries may influence mothers who visit ANCs frequently to opt for caesarean deliveries [10]. Another possible reason could be that ANC visits help in early identification of complicated cases which then lead to more C-Sect. [51], although our analysis did not demonstrate a rise in pregnancy complications across the five years studied. The association between respondents’ education levels and C-section has been found to be significant in several studies in other LMICs [52–54]. Some studies report that better-educated women prefer C-sections as they view them as being safer, causing less pain, interfering less with work and leisure, and being socially more prestigious than vaginal delivery [55, 56].

Women who have previously suffered a pregnancy loss have higher odds of C-sections as they may perceive this as a safe option to deliver a “precious baby” [57] conceived after earlier miscarriages or via assisted reproductive technologies.

### Chhattisgarh and Tamil Nadu – a comparison

When compared to CG, C-section prevalence is far higher in TN across both NFHS-4 (9.9% vs. 34.1%) and NFHS-5 (15.2% vs. 44.9%). TN has demonstrated a very high prevalence of C-sections (up to 45%) as far back as 1997 [58]. A closer study of the contrast between Chhattisgarh and Tamil Nadu in 2019-21 yields some interesting insights. There are striking differences in the proportion of women in CG and in TN respectively who belonged to Scheduled Tribes (43.8% vs. 1.7%), who had received a higher education (9.6% vs. 40.3%), who lived in urban areas (16.7% vs. 40.4%), and who belonged to the “non-poor” category (37% vs. 82.8%) (Table 2).

Other studies have demonstrated a lower rate of C-sections among tribal women due to differential access to health services [59, 60]. Higher levels of education have been demonstrated to increase C-section rates [14, 61]. A 42-country study [62] found that C-section prevalence was highly uneven across socioeconomic status – it was below 1% among the poorest quintile in 20 countries, and below 1% for 80% of the population in 6 countries. Only in 5 countries did the C-section rates amongst the poorest exceed 5%. Several studies report higher C-section rates among the rich [10, 61, 63]. Higher levels of urbanisation are associated with a greater prevalence C-Sects [61, 64]. Lower fertility rates and greater concern for the safety of the child and the mother led to higher C-section rates in urban areas even in the absence of clinical need [65]. These factors all could conceivably contribute to the difference between C-section prevalence in CG and in TN.

**Table 9:** Differences in Health Infrastructure – CG and TN (2021)

State	Population (2011) in crores	District Hospitals (DHs)	Sub-District Hospitals (SDUs)	Community Health Centres (CHCs)	Urban CHC	24 × 7 functional CHCs against requirement (%)	Obstetricians & gynaecologists - vacancy against sanctioned in rural areas (vacancy %)
CG	2.55	23	3	31	4	170/212 (80%)	129/167 (77%)
TN	7.21	32	152	367	11	385/385 (100%)	24/61(39%)

Sources: Rural Health Statistics, Ministry of Health and Family Welfare [67], Health Dossier 2021 [68, 69]

The availability and accessibility of comprehensive obstetric care facilities play a critical role in providing C-section deliveries [66]. Table 9 demonstrates the considerable difference in the number of First Referral Units^1^, District and Sub-District Hospitals between both the states that is not commensurate with their respective populations. Tamil Nadu is lauded as a model for public health care delivery in India with 100% of the prescribed number of Community Health Centres (CHCs) functional and a 39% shortfall in the sanctioned number of obstetricians and gynaecologists, while Chhattisgarh has 80% of the required CHCs and a 77% shortfall. In addition, there is a significant rural-urban gap and regional variations in the utilisation of maternal and child health services such as ANC visits, immunisation and institutional delivery, with a higher utilisation of these services in TN as compared to CG [70, 71]. We conclude that the difference in availability of health facilities and infrastructure in CG and in TN could be another possible reason for the differential C-section rates between these states.

Three need factors for C-section deliveries are compared across CG and TN – (a) the presence of pregnancy complications [61], (b) avoidable high-risk fertility behaviour (mother’s age < 18 years or > 34 yrs., birth interval lesser than 24 months or birth order > 3 - each of these high-risk factors alone or any of them in combination could result in adverse birth outcomes) [72, 73] and (c) obesity (BMI > = 30) [13, 74].

**Table 10:** Need Factors for C-section Deliveries, NFHS-5

NFHS-5	India	CG	TN
Pregnancy Complications	39.5	33.2	30.7
High-Risk Fertility Behaviour	33.1	27.0	19.7
Obesity	4.7	2.09	13.8
C-section Prevalence	21.5	15.2	44.9

Despite the fact that both pregnancy complications and high-risk fertility behaviour were more prevalent in CG, TN had the higher prevalence of C-sections (Table 10). Obesity was far more prevalent in TN, and has been found to be a significant risk factor for C-section in several studies [13, 74]. However, whether this alone contributes to the nearly 3-fold difference in C-section prevalence between the two states is debatable.

### Place of delivery and wealth inequalities

A number of studies have concluded that the place of delivery (public or private) is the single most important predictor of whether the birth is vaginal or via C-Sects [21, 75–78]. Our study corroborates this. In Chhattisgarh, a woman is ten times more likely to undergo a C-section in a private hospital than in a public hospital. This could be due to a lack of adequate or high-quality services in public health institutions – only 9.7% of births in public facilities were by C-section.

Trends in C-section prevalence by economic status and type of healthcare facility in the Middle East and North Africa showed that C-sections are higher among the rich and in private health facilities [79]. This could because affluent women have fewer financial constraints compared to their poorer counterparts. However, women belonging to the richest quintile in Ghana still underwent the most C-sections in spite of the procedure being covered under the country’s free maternal care policy. Other costs associated with transport and services might be the reason for these persisting inequalities [80].

The inversion in income-based inequality in Tamil Nadu in private institutions in 2019-21, with a greater proportion of the poor than the non-poor delivering via C-section, is difficult to explain. Comparable trends have been observed in some developed nations such as Italy and France, where women who were less educated and presumably from worse socioeconomic backgrounds were more likely to undergo C-Sects [81, 82]. One factor might be that classified by national standards, only 17% in Tamil Nadu fall in the lowest two quintiles and are “poor”, and upon this low denominator, there might be a larger number of women with underlying medical complications that require C-sections. Preliminary analysis indicated that this is not so, and that delivery and pregnancy complications were *not* more prevalent among the poor that delivered in private facilities compared to the non-poor in TN. This leads us to the conclusion that the inversion may not be because those of the poor who go to private facilities do so because their health is worse in terms of pre-conditions requiring C-sections. We conclude that other reasons are at play, which might come to light upon further study.

### Policy implications

Several policy interventions have been advocated and tested with varying degrees of success. Clearer guidelines defining when a C-section can be performed have been found to reduce rates [83] but are difficult to enforce as providers need to use discretion during diagnosis [84]. In order to measure, track, and compare caesarean section rates over time and between healthcare facilities, the WHO suggests using the Robson classification system, and proposes detailed guidelines for its usage, implementation, and interpretation, including standardisation of words and meanings [33]. Mandating a smaller difference in fees between C-sections and vaginal deliveries might result in providers preferring the less time-consuming and safer (in the short-term) option of C-sections. A global obstetric fee was introduced in Australia but was withdrawn in 1995 when it was found ineffective in deterring the increase in C-section rates [85]. This option is, in addition, not viable in the Indian context, where fees for services are common. Public dissemination of the adverse effects of C-section rates in each hospital has also been suggested as an intervention which might tie in with greater awareness and help bring down C-section prevalence. There is a paucity of rigorous studies on the effect of mass media campaigns on unnecessary C-Sect [86]. One study which did evaluate these effects found that pregnant women’s understanding of and intentions towards vaginal births improved in the short term but warranted further research on long-term consequences [87].

We recommend, overall, that threshold levels for C-sections be applied cautiously, as several inter-category variations exist, and in states at advanced levels of demographic transition, need factors for C-sections may be more prevalent. There is an alarmingly high proportion of poor women undergoing C-sections in the private sector in Tamil Nadu. This requires further analysis and corrective action in case some of these are clinically unnecessary. Increasing access to 24 × 7 health infrastructure in Chhattisgarh, especially to disadvantaged groups, would help close the gap between the poor and the rich as well as between tribes as non-tribes.

This study brings fresh insights into the increasing prevalence of C-sections according to the new NFHS-5 data, compared with prevalence in the previous round. We highlight the new findings of this paper below:

- We have looked at new data from NFHS-5 (2019-21) and have studied trends in C-section rates over the 5-year period since the last round of NFHS. We have brought out the contrast between two states in India with very different demographic and developmental features - a detailed analysis of the differences between demographic, economic and infrastructural factors in Chhattisgarh and Tamil Nadu, and their possible effects on C-section rates has been conducted.
- We have, in addition, analysed inequalities across wealth relating to delivery by C-section.
- The place of delivery had the greatest impact on whether or not delivery was by C-section, implying that need factors are not necessarily the key reason for surgical deliveries.
- In Tamil Nadu, there was a surprising inversion in private sector hospitals, with the poor more likely to deliver via C-sections, which was not related, upon primary analysis, to a higher prevalence of need factors.

### Limitations

The limitations of the study are, primarily, those that are inherent to the NFHS datasets: (a) as NFHS collects cross-sectional data, establishing causal inferences between C-section delivery and independent variables is tenuous; (b) it is difficult to definitively ascertain the reasons for the increasing rates of C-sections because NFHS doesn’t capture data on whether these were performed due to medical or non-medical reasons; (c) data on whether the previous delivery was a C-section is not captured, so it is not possible to estimate what proportion of the C-sections can be attributed to this; and (d) caesarean deliveries can be influenced by many cultural, physiological, and behavioural factors; however, we could not include these factors in the analysis due to the unavailability of information in the dataset.

## Conclusion

This paper brings to light the degree of disparity in C-section prevalence between private and public-sector hospitals across the country and in TN and CG, and points out the trends over the five-year interval between the two rounds of NFHS studied. The rise in prevalence of C-sections despite a decrease in the presence of pregnancy complications suggests that factors other than medical ones influence the mode of delivery. The place of delivery is the most significant factor influencing C-section deliveries as an outcome, far exceeding the presence of pregnancy complications, maternal obesity and age. How much of this high prevalence of C-sections in the private sector is attributable to economic incentives, maternal request, risk aversion or other non-medical factors needs to explored. We need effective policy and regulatory frameworks as well as public education to reduce the adverse medical and financial impacts of unnecessary caesarean deliveries.

## Data Availability

The datasets generated during and/or analysed during the current study are available in the DHS and IIPS website, accessible at https://dhsprogram.com/data/available-datasets.cfm and http://rchiips.org/nfhs/districtfactsheet_NFHS-5.shtml respectively.

## Footnotes

1 With the aim of strengthening referral services and catering to the expected increase in demand for health services, the National Health Mission has set up First Referral Units (FRUs) that provide 24-hour services at the Community Health Centre (CHC) level. FRUs provide specialist care in Medicine, Obstetrics and gynaecology, Surgery and paediatrics.
