# Correction: Variations in the prevalence of caesarean section deliveries in India between 2016 and 2021 – an analysis of Tamil Nadu and Chhattisgarh

**DOI:** 10.1186/s12884-023-06020-7

**Published:** 2023-09-26

**Authors:** Varshini Neethi Mohan, P Shirisha, Girija Vaidyanathan, V R Muraleedharan

**Affiliations:** Department of Humanities and Social Sciences, Indian Institute of Technology Madras (IIT Madras), Chennai, 600 036 Tamil Nadu India

**Correction: BMC Pregnancy Childbirth 23, 622 (2023)**

10.1186/s12884-023-05928-4

Following publication of the original article [1], the authors identified an error in the Abstract, under Background section.

The sentence currently reads:

The prevalence of C-sections in India increased from 17.2% to 2006 to 21.5% in 2021.

The sentence should read:

The prevalence of C-sections in India increased from 17.2% in 2016 to 21.5% in 2021.

The original article [1] has been corrected.

## References

[1] Neethi Mohan V, Shirisha P, Vaidyanathan G (2023). Variations in the prevalence of caesarean section deliveries in India between 2016 and 2021 – an analysis of Tamil Nadu and Chhattisgarh. BMC Pregnancy Childbirth.

